# Decreased Activation of Subcortical Brain Areas in the Motor Fatigue State: An fMRI Study

**DOI:** 10.3389/fpsyg.2016.01154

**Published:** 2016-08-03

**Authors:** Li J. Hou, Zheng Song, Zhu J. Pan, Jia L. Cheng, Yong Yu, Jun Wang

**Affiliations:** ^1^College of Physical Education and Sports, Beijing Normal UniversityBeijing, China; ^2^State Key Laboratory of Cognitive Neuroscience and Learning, Beijing Normal UniversityBeijing, China; ^3^Department of Kinesiology, Mississippi State University, StarkvilleMS, USA

**Keywords:** motor fatigue, sensorimotor areas, thalamus, basal ganglia, fMRI

## Abstract

One aspect of motor fatigue is the exercise-induced reduction of neural activity to voluntarily drive the muscle or muscle group. Functional magnetic resonance imaging provides access to investigate the neural activation on the whole brain level and studies observed changes of activation intensity after exercise-induced motor fatigue in the sensorimotor cortex. However, in human, little evidence exists to demonstrate the role of subcortical brain regions in motor fatigue, which is contradict to abundant researches in rodent indicating that during simple movement, the activity of the basal ganglia is modulated by the state of motor fatigue. Thus, in present study, we explored the effect of motor fatigue on subcortical areas in human. A series of fMRI data were collected from 11 healthy subjects while they were executing simple motor tasks in two conditions: before and under the motor fatigue state. The results showed that in both conditions, movements evoked activation volumes in the sensorimotor areas, SMA, cerebellum, thalamus, and basal ganglia. Of primary importance are the results that the intensity and size of activation volumes in the subcortical areas (i.e., thalamus and basal ganglia areas) are significantly decreased during the motor fatigue state, implying that motor fatigue disturbs the motor control processing in a way that both sensorimotor areas and subcortical brain areas are less active. Further study is needed to clarify how subcortical areas contribute to the overall decreased activity of CNS during motor fatigue state.

## Introduction

The neural mechanism of motor fatigue is still unclear although this phenomenon has been studied for almost half a century (Bigland-Ritchie and Lippold, 1979). In the early works, most studies focused on the physiological changes of the muscles (Bigland-Ritchie et al., 1986; Gandevia, 1988; Pagala et al., 1991), such as run-down of the energy reserves, accumulation of blood lactate, unbalance of H+, as well as increased free radical content (St Clair Gibson et al., 2005; Reid, 2008). Therefore, motor fatigue was primarily referred to as muscular fatigue. However, accumulating evidences reveal that the supraspinal structures also exhibit some changes of activity under motor fatigue state, especially the motor cortex of the central nervous system (CNS; Taylor et al., 1996, 2006; Gandevia, 2001), whose output is not sufficient to drive the muscle maximally under motor fatigue state (Jones and Hunter, 1983; Enoka, 1995; Taylor et al., 2000; Amann and Dempsey, 2008).

Cortical sensorimotor areas play a dominant role in human motor control. Recent studies found that there was a linear relationship between neuronal activity in several brain areas (i.e., the primary motor cortex and the cerebellum) and the sum of EMG activity of various hand muscles in monkeys (Townsend et al., 2006; Bourguignon et al., 2013). Our previous human study also shows that the change of activation level in cortical brain areas is related to motor fatigue (Hou et al., 2012). Also, there is evidence showing that CNS has to increase its drive to relevant motor neuron pools or increase the firing frequency of the already active units to increase muscle force production, as is demonstrated by an increase in the electromyography (EMG) activity (Jones and Hunter, 1983).

The subcortical brain regions, such as the thalamus and the basal ganglia, play critical roles in the regulation of motor function (Buot and Yelnik, 2012). By using the electrophysiological recording technique, we observed the variation of neuron activity in striatum and found that the percentage of high frequency neurons is significantly increased, and the amounts of bursting style neurons are increased in striatum during motor fatigue (Qiao et al., 2010).

Inconsistent findings were reported regards change of brain activation in response to motor fatigue. Dettmers et al. (1996) show that motor fatigue dose not induce changes in the activation intensity of the primary sensorimotor areas, SMA, or basal ganglia, while the only changed activation was observed in a small cluster in the dorsolateral prefrontal cortex. In contrary, other studies found increase in the number of activation voxels in the sensorimotor cortex, SMA, cerebellum, and dorsolateral prefrontal cortex in motor fatigue condition (Liu et al., 2003).

Brain image studies observed associations between motor fatigue and altered neural activation on the cortical level, indicating involvement of the CNS in motor fatigue (Gandevia, 2001; Taylor et al., 2006; Hou et al., 2012). To further our understanding of how the CNS acts in response to motor fatigue, the present study aims to exam how the activation of the sub-cortical areas changes when people experience exercise-induced motor fatigue. To this end, we recruited a group of healthy subjects to measure (1) the changes of activation in sub-cortical areas, i.e., thalamus and basal ganglia, when performing simple motor tasks before and under exhaustive exercise, which induces motor fatigue state with well-control procedures, and (2) the changes of activation in the sensorimotor areas, in which inconsistence observation of activation changes have been made.

## Materials and Methods

### Subjects

Eleven healthy right-handed subjects participated in this study after signing informed consents (seven male, four female; age 23.2 ± 3.5 years). All subjects underwent the comprehensive verbal screening procedure to ensure that they do not violate any of the exclusive criteria for functional magnetic resonance imaging (fMRI) experiment: (1) history of neurological or cardiovascular disease; (2) medications; (3) cochlear implants or any metal objects in the body; (4) cardiac or neural pacemakers; and (5) history of musculoskeletal injury in both lower limbs. The Ethics Committee of Beijing Normal University approves all experimental procedures.

### Exhaustive Exercise Protocol

**FIGURE 1:**
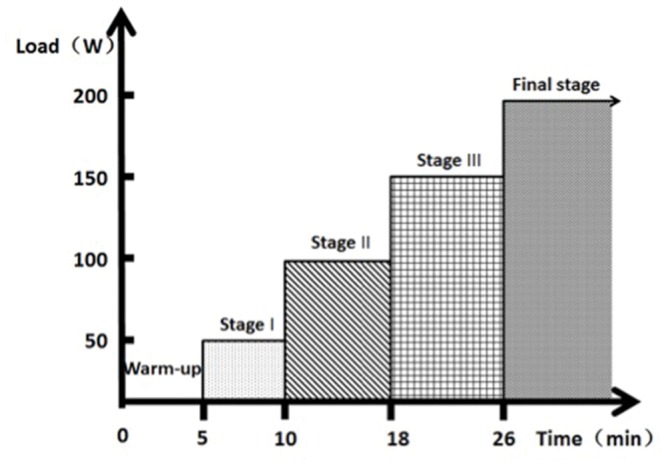
**Protocol of exhaustive exercise.** The process begins from a 5 min warm-up, 5 min stage I, 8 min stage II, 8 min Stage III, and a certain duration of final stage according to the physical ability of the participants.

In order to reach the motor fatigue state, participants performed the exhaustive exercise protocols on a cycle ergometer (Monark 834 E, Sweden). Heart rate was measured every 5 s using short-range radio telemetry (S725XPolar, Finland). A portable lactate analyzer (YLS9-Lactate scout, German) was used to analyze all blood samples extracted from the finger sticks. Participants completed a 5-min warm-up with no resistance at a cadence of 50 rev min^-1^. At the conclusion of the warm-up, 1 kg of resistance is added, yielding a power output of 50 W. Power output increases to 100 W at the second stage, to 150 W for the third stage and 200 W for the final stage. Participants pedaled at a constant cadence of 50 rev min^-1^ until reaching exhaustive state (**Figure 1**). The state of motor fatigue is defined as the time point when the participant cannot maintain the cadence and the heart rate arrives 90% of the maximum heart rate.

### Experiment Design

**FIGURE 2:**
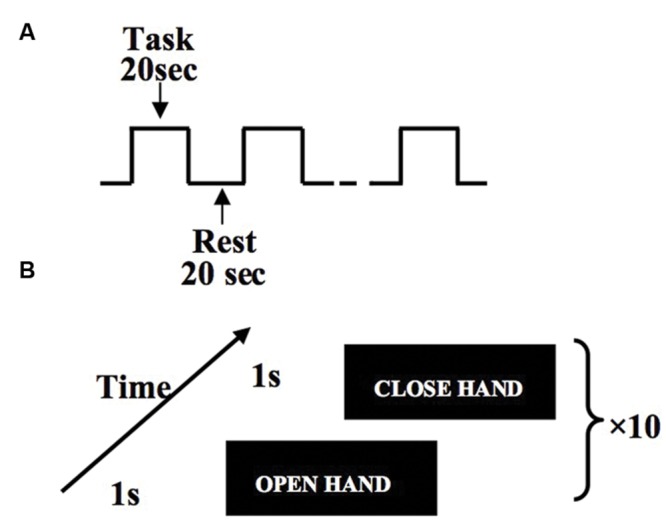
**Experiment design of hand flexion and extension movements.** Within the block design, task blocks and rest blocks are arranged in alternative order, and each block lasts 20 s **(A)**. Within each task block, subjects perform one hand flexion/extension every 2 s **(B)**.

After subjects reached the motor fatigue state, they were guided to the MRI scanner. Subjects performed hand flexion and extension movements according to the instructions reflected in the mirror from a computer screen. For every subject, we controlled the delay to be approximately equal between the exhaustive exercise and the MRI acquisition. Subjects were guided to the scanner right after the physical measurement. Each run of MRI scanning includes 10 trials with each trial lasting 20 s (**Figure 2A**). Within each task trial, there are 10 hand open and 10 hand close movements, which are guided by visual signals (**Figure 2B**).

### MRI Data Acquisition

The MR protocol was performed using 3 Tesla whole-body system (Siemens, Erlangen, Germany) using blood oxygen level-dependent (BOLD) fMRI. The head of the subject was immobilized using foam cushions and tape, with their ears plugged. The protocol included: (i) one sagittal T1-weighted image to localize functional and anatomical axial slices; (ii) 33 axial gradient echo-planar images (EPI; 3.8 mm, gap 0.7 mm, TR = 2 s, TE = 30 ms, bandwidth = 2520 Hz/pixel, Flip angle = 90°, FOV = 218 mm × 218 mm, in-plane resolution = 64 mm × 64 mm). The whole protocol lasts 40 min.

### Functional MRI Data Analysis

Functional images were preprocessed using SPM5 software (Wellcome Department of Cognitive Neurology, London, UK) and Matlab. Before the preprocessing, the first five time points were discarded to avoid the disequilibrium in the magnetic field in the beginning of the scan. Images of each subject were realigned by using the first slice as the reference, and then normalized into the MNI space (Montreal Neurological Institute) using the template provided by SPM. At last, the 3 mm × 3 mm × 3 mm functional images were spatially smoothed with a Gaussian filter (4 mm × 4 mm × 4 mm full-width at half-maximum).

After the above preprocesses, functional images were first analyzed on the individual level to determine the activation volumes in response to hand movement. According to our block design, the boxcar waveform of the sustained activity of simple hand movement was convolved with the theoretical hemodynamic response, for both before and after exhaustive exercise conditions. Then, on the group level, 2-tailed one sample *t*-test analysis was performed based on the outcome of each subjects’ statistical parameter maps from the individual level analysis. Those volumes, which pass the significance threshold (*p* < 0.05) and had cluster size larger than 54 voxels, were reported as activation volumes. In the end, we compared the activation differences before and under motor fatigue state. Only the voxels that are activated in both before and under motor fatigue state conditions were included in this contrast. To do that, a union mask was generated from the significant positive activation maps of the two groups.

## Results

**FIGURE 3:**
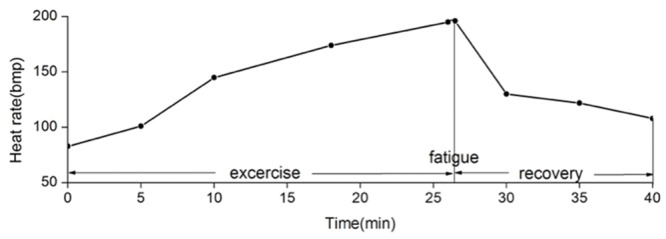
**Mean heart rate changes during and after exhaustive exercise.** We define that the state of motor fatigue is the time point when the participant cannot maintain the cadence, and when heart rate arrives 90% of the maximum heart rate. In this experiment, participants reach the fatigue state after 26 min of exhaustive exercise in average.

All subjects reached motor fatigue state after the exhaustive exercise (**Figure 3**).

### Brain Activation Maps before and during Motor Fatigue State

**FIGURE 4:**
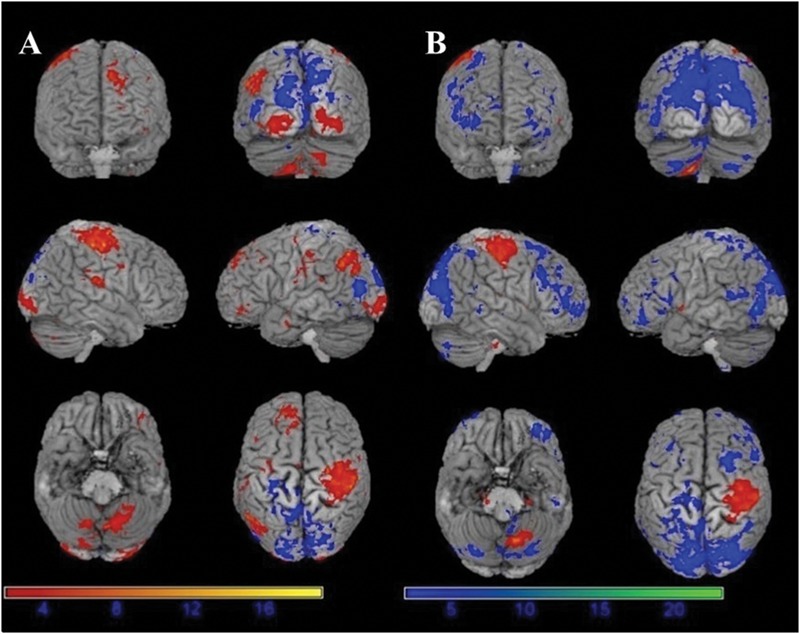
**Significant activation (red) and deactivation (blue) volumes when subjects are performing hand movement before **(A)** and during **(B)** motor fatigue.** Color bar: *t*-values.

The motor task evoked significant activations in the following brain areas in both before and under motor fatigue state conditions: the bilateral sensorimotor area, the premotor area, the supplementary motor area, the cerebellum, and the basal ganglia (**Figure 4**).

### Differences on Activation Volumes before and under Motor Fatigue State

**FIGURE 5:**
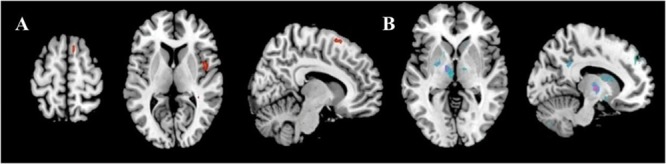
**Activation volumes show significant differences before and after exhaustive exercise. (A)** After > before; **(B)** Before > after.

The results showed the intensity and size of activation volumes of ipsilateral thalamus and striatum significantly decreased during the motor fatigue state as compared to before the motor fatigue state. Moreover, the size of activation volumes in contralateral sensorimotor area was smaller (see in **Table 1**, sensorimotor area, Nb Vx), but there was no significant difference in the strength of activation (**Figure 5**; **Table 2**). It is worth noting that there was also difference between the extending of two activation maps. When the subjects perform the simple motor task under motor fatigue, the ipsilateral sensorimotor area was not as active as it was before, indicating the CNS tends to recruit less cortical and subcortical volumes when performing movements under motor fatigue.

**Table 1:** Significant^†^ activation volumes before and during motor fatigue state.

Region		Before			*t*	BA^∗^	NbVx^∗∗^		During			*t*	BA	NbVx
		*x*	*y*	*z*					*x*	*y*	*z*			
Sensorimotor	R	45	-15	57	17.65	4	72	R	42	-18	51	12.10	4	66
	R	51	-21	57	13.43	3,2,1	154	R	42	-24	57	9.17	3,2,1	136
SMA	R	6	-3	60	6.16	6	108	R	9	9	63	3.52	6	56
	L	-6	-3	60	7.47	6	77	L	-9	-3	63	4.03	6	42
PM	R	24	-12	69	16.19	6	260	R	36	-12	57	18.70	6	101
	L	-42	0	54	4.43	6	40							
Cerebelum_6	R	27	-57	-24	16.82	37	174	R	27	-63	-18	6.01	19	76
	L	-30	-51	-24	14.05	37	210	L	-9	-60	-15	10.27	18	171
Thalamus	R	18	-15	3	17.65	NA	171	R	6	-27	3	7.04	NA	82
	L	-9	-9	9	5.19	NA	72							
Putamen	R					NA	144	R					NA	85
	L					NA	72							
Caudate	R					NA	40	R					NA	9
	L					NA	84							
Pallidum	R					NA	25	R					NA	11
	L					NA	18							
Insula	R	44	5	6	3.39	48	65	R	45	3	6	9.53	48	61
	L	-44	-3	9	4.15	48	31	L	-42	-3	3	4.99	48	31
postcentral	L	-45	-21	33	7.61	3	107							
parietal_inf	L	-51	-21	42	6.99	40	23							
Rolandic_Oper								R	45	-3	9	2.93	48	56
								L	-45	-6	3	12.10	48	39
supraMarginal	L					40	68	R	48	-33	28	5.13	48	47
temporal_sup	L	-51	-30	21	7.34	48	39	R	57	-18	12	5.70	48	26
frontal_sup	L	-18	54	30		9	185							
frontal_sup_medeal	L	-9	39	57		9/8/10	43							
Frontal_Mid_Orb	L	-39	48	-9		10	29							
Frontal_Inf_Orb	L					11	52							
Frontal_Inf_Tri	L	-45	39	6		46/47	25							
Temporal_Mid	L	-57	-3	-18		21	78							

*^†^*P* < 0.05, cluster size > 54. ^∗^BA, Brodmann area, ^∗∗^Nb Vx, number of voxels. Abbreviations: SMA, supplementary motor area; PM, premotor area; inf, inferior; Oper, Operculum; sup, superior; Mid, middle; Orb, orbital; Tri, triangularis.*

**Table 2:** Brain volumes showing significantly^†^ different level of activation before and under fatigue state.

	Region	Hemisphere	*x*	*y*	*z*	*t*
Before > after	Thalamus	L	-12	-9	0	4.66
	Striatum	L				
After > before	SMA	R	9	15	63	3.14
	Insula	R	45	3	6	4.17
	Hippocampus	R	33	-30	-6	3.29
	Cerebellum	R	39	-42	-33	3.48

*^†^*P* < 0.05, cluster size > 54.*

## Discussion

From this experiment, we found that motor fatigue affects not only the neural excitability on the cortical level (i.e., sensorimotor cortex and the SMA), it affects also the subcortical brain regions (i.e., thalamus and striatum). This finding has not been reported systematically in previous studies.

Previous neurophysiological studies found that, when under motor fatigue, the output of cortical sensorimotor areas may not be adequate to drive the motor neurons to produce maximal movement force (Jones and Hunter, 1983; Enoka, 1995). This finding is supported by recent studies using transcranial magnetic stimulation (TMS), which find that some force loss in fatigue may be due to inadequate descending drive from the motor cortex (Taylor and Gandevia, 2001). The present study found that neural activation in the sensorimotor cortex and the SMA declined when people under moto fatigue. This finding provides neural image supports for the notion that the cortical areas play role in motor fatigue.

**FIGURE 6:**
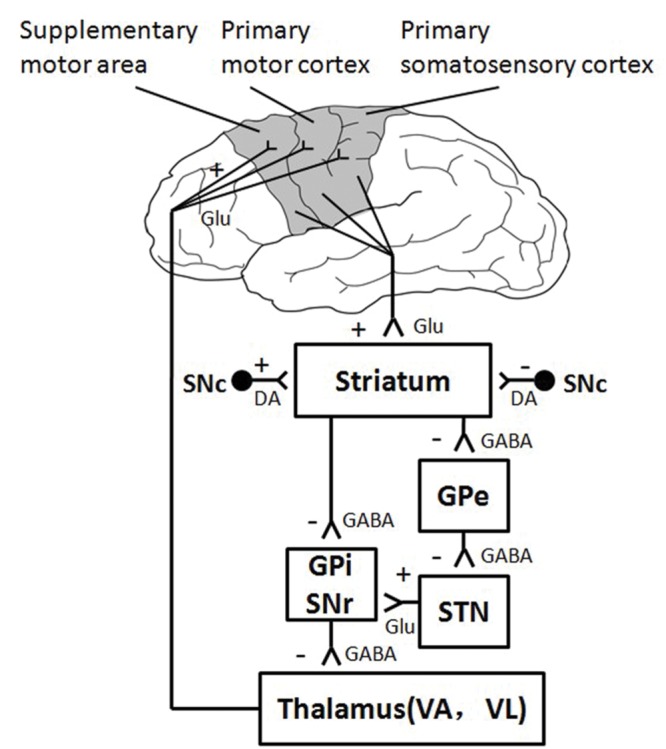
**Direct and Indirect Pathway involved in Motor Control**.

The thalamus and the striatum are two important subcortical brain areas, which participate in human motor control by interacting with the cerebral cortex through parallel neural circuits (Alexander et al., 1986; Baston and Ursino, 2015). Recent diffusion weighted imaging and tractography study in human find a regional network among SMA, subthalamic neucleus (STN), and the inferior frontal cortex (IFC; Aron et al., 2007). Striatum, more specifically the STN, is the input station of the basal ganglia that receive excitatory afferent input from the cortical motor areas, such as the sensorimotor cortices and SMA (Prodoehl et al., 2009). The thalamus receives inhibitory signals from the output station (i.e., Gpi and SNr) of the basal ganglia, and it sends excitatory output to cerebral cortex in turn (**Figure 6**). The present study found decreased neural activation in the subcortical structures of thalamus and striatum, indicating that the subcortical areas involve in motor fatigue as well. In other words, motor fatigue seems be associated with declined neural activation on both cortical and subcortical levels. Although the excitatory and inhibitory circuits between striatum and thalamus is beyond the exploration of this experiment such that we are not able to infer the temporary changes of activation from cortical region to thalamus, from above mentioned knowledge, we can still make the preliminary conclusion that motor fatigue state influences the neural substrates of simple grasp movement. This effect exhibits as an overall decrease of activation of the corticostriatal and cortico-thalamic pathways. It is highly possible that the decreased activation of sensorimotor areas may account for the decreased activation of striatum, and the decreased activation of thalamus may contributes the loss of activation of sensorimotor areas.

We want to clarify that the present study did not monitor the force and movement frequency produced by finger movements due to technical limitation associated with MRI scanning. As a consequence, we cannot ascertain if the decreased cortical and subcortical activation observed would attribute to the possible reduction of motor performance. In other words, the present study found an association between motor fatigue and the CNS, while future studies are needed to further exam the proposed causal relationship between the less activated cortical and subcortical areas and the declined motor performance under fatigue state. We also want to point out that subjects were asked to evaluate their own performance in the study. In general, subjects reported be able to follow the pace and range of the movement as required in both conditions. However, the self-report alone is not sufficient to present the real change of movement performance due to fatigue. Moreover, studies have shown that there could be a motor fatigue associated over-estimation of force of muscle contraction (Jones and Hunter, 1983). Therefore, we still cannot rule out the possibility that the reduced activity on the cortical and subcortical level is partially responsible for reduced muscular contraction under fatigue.

Recent study had found that striatum is highly relevant with motor and cognitive function. Our knowledge of striatum’s role in motor control mainly came from some common neurodegenerative disease, such as Parkinson disease (PD), Huntington disease (HD), Tourette syndrome (TS), etc. Patients with these diseases exhibit motor deficits and always show some structural abnormality of striatum. The disturbance of striatum activation during motor fatigue state might also interrupt with the motor and cognitive function; further experiment is needed to examine how motor fatigue effect motor and cognitive functions.

## Conclusion

We used exhaustive physical exercise to induce motor fatigue state; then, under the effect of motor fatigue, the neural response of simple movements exhibiting dramatic differences compared with before the exhaustive physical exercise. We observed an overall decrease of activation in sensorimotor areas, SMA, as well as in the thalamus and basal ganglia.

It is well known that the cortical motor areas have anatomical connection with the subcortical brain regions, and these connections forms complex excitatory and inhibitory circuits (Alexander et al., 1986). The interference of these neural circuits leads to various pathological conditions (Shepherd, 2013). The result from this experiment revealed for the first time the overall changes of these circuits in response to motor fatigue; however, the contribution of each brain regions is still not clear. We propose that further study should be deployed at two directions, the role of basal ganglia in central motor fatigue and the effect of motor fatigue on patients with various movement disorders.

## Author Contributions

LH and JW conceived and designed the study. YY and JC performed the experiments. ZS and LH analyzed the data. ZS and ZP wrote the paper. LH, ZS, and ZP reviewed and edited the manuscript. All authors read and approved the manuscript.

## Conflict of Interest Statement

The authors declare that the research was conducted in the absence of any commercial or financial relationships that could be construed as a potential conflict of interest.

The reviewer QL and handling Editor declared their shared affiliation, and the handling Editor states that the process nevertheless met the standards of a fair and objective review.
